# Improvement and Quantification of Extraction Methods for Annual Bluegrass Weevil Larval Populations

**DOI:** 10.3390/insects16090986

**Published:** 2025-09-22

**Authors:** Albrecht M. Koppenhöfer, Olga S. Kostromytska, Ana Luiza Sousa

**Affiliations:** Department of Entomology, Rutgers University, 96 Lipman Dr., New Brunswick, NJ 08901, USA

**Keywords:** annual bluegrass weevil, extraction, sampling, monitoring, turfgrass

## Abstract

The annual bluegrass weevil (ABW), *Listronotus maculicollis* (Kirby), is a significant pest of short-mown turfgrass in eastern North America. Proper monitoring of this pest may reduce insecticide applications. We investigated the extraction rate and labor involved in modifications of the two currently available methods for sampling ABW larvae: submersion of turf cores in saturated saline solution and heat extraction of cores on modified Berlese funnels. Using 5.7 cm diameter cores, submersion extracted 1.8× more larvae in saline solution than in water. Among the salt extraction variants, splitting the cores into four pieces before submersion was the best compromise between extraction rate and time requirement. Using intact cores extracted 40% fewer larvae while taking 18% less time, whereas destructive searching cores before submersion extracted 24% more larvae but required 64% more time. Using smaller cores (3.5 cm diam) took 18% less time and extracted 23% more larvae, but required more time sampling in the field. Larval stage averages did not differ significantly between salt extraction variants. Heat extraction, including destructively searching the desiccated core, extracted 60% more larvae but required 87% more time than four-piece salt extraction. Excluding the desiccated core, heat extracted as many larvae as four-piece salt extraction and required 16% less time. However, heat extraction requires three to four days and space that can be kept at around 32 °C. The method of choice for ABW larval extractions depends on whether the sampler prefers a high extraction rate, less labor, or quicker results, and whether space for heat extractions is available.

## Simple Summary

The annual bluegrass weevil is a significant turfgrass pest in eastern North America. There are two methods for sampling ABW larvae: submersion of turf cores in saturated saline solution and heat extraction of cores, but their extraction rates and labor involved have not been quantified. Among salt extraction variants, splitting the cores into four pieces before submersion was the best compromise between extraction rate and time requirement. Using intact cores extracted 40% fewer larvae while taking 18% less time, whereas destructive searching cores before submersion extracted 24% more larvae but required 64% more time. Heat extraction, including destructively searching the desiccated core, extracted 60% more larvae but required 87% more time than four-piece salt extraction. Excluding the desiccated core, heat extracted as many larvae as four-piece salt extraction and required 16% less time. However, heat extraction requires four additional days to determine larval densities and space that can be kept at around 32 °C.

## 1. Introduction

Low damage tolerances in turfgrass, especially on short mown golf course turfgrass, have promoted preventive insecticide applications, which are made before the need for applications can be assessed through sampling [1,2,3,4]. In the case of highly destructive and difficult to manage insect pests like the annual bluegrass weevil (ABW), *Listronotus maculicollis* (Kirby), this can lead to excessive insecticide use and ultimately to insecticide resistance, which in ABW is already very common and widespread and often broad-spectrum in nature [5,6,7].

The ABW has 2–3 generations per year with multiple life stages present simultaneously; asynchrony of stages increases during the growing season [1,3,4]. In early spring, overwintered adults migrate back to short-mown turfgrass areas to feed and mate and oviposit in the grass stems [8]. The first three larval stages feed inside the stem but cause only limited damage. Severe damage is caused by the fourth and fifth stage larvae that feed from shallow burrows in the soil/thatch layer on the base of stems and on the crowns. Pupation occurs near the soil surface.

Even though the adults do not cause any significant damage, many control efforts target them before they can lay eggs, particularly in spring when they are the only stage present. However, against insecticide-resistant ABW populations, the available adulticides, pyrethroids and chlorpyrifos, are less effective or completely ineffective depending on the degree of resistance [9]. In contrast, there are still several larvicides that ABW are either minimally or not resistant to [9] (AMK, unpublished data). However, the mechanism primarily responsible for the often broad spectrum of resistance in ABW populations is enhanced enzymatic detoxification [10]. It is therefore likely that currently still effective insecticides, if overused, will eventually also lose efficacy.

Central to effective mitigation and delaying of insecticide resistance development in insect populations are monitoring and sampling methods with high predictive power and ease of use, and that allow the commodity manager to restrict insecticide application to only when and where necessary [2,4]. Several methods are currently available for monitoring adult ABW, including clippings examination, vacuuming, and soap flushing [1,3,4]. Sousa et al. (2023) [11] found that soap flushing was the most effective and reliable method, and through modifications in soap concentration, application volume, and application interval, optimized the extraction efficiency to >80%. However, adult densities are only an indirect predictor of damage potential since significant damage is not caused by the adults. Moreover, many factors may affect how many larvae will result from a given adult density, including grass species composition, soil moisture, temperature, mowing height, and potentially other factors.

Larval monitoring has greater predictive power since larvae are the stage that causes significant damage. However, the estimate for the damage threshold is rather wide at 30–80 ABW larvae per 0.093 m^2^ (square foot) [1,3,4] because the damage potential by a given larval density may also be affected by the same above factors. Currently, there are two methods for sampling ABW larvae: submersion of turf cores in salt water and heat extraction of turf cores. These methods are widely used by scientists and consultants, but not commonly by golf course superintendents. However, their extraction rates (i.e., number of larvae extracted) have not been determined, nor have attempts been made to optimize these methods and quantify the labor and time involved. Such knowledge would help scientists and consultants choose the best extraction method for their purpose and may lead to wider use among superintendents.

The goal of this research was to optimize the salt extraction method by comparing different degrees of breaking up the turf cores before extraction and using different-sized cores. The extraction rates of the different salt extraction method variants and the time required for the extraction were compared to those of heat extraction. We also determined the effect of larval stage mix on the extraction rate of the best salt extraction variant and of heat extraction. Lastly, we tested the effect of splitting cores on the extraction rate of heat extraction.

## 2. Materials and Methods

### 2.1. General Methods

Soil/sod cores (5.7 cm diameter × 4 cm depth unless described otherwise) for the extraction experiments were taken with a tubular turf plugger (Turf-Tec International, Tallahassee, FL, USA) from fairway areas with a history of ABW infestations at Pine Brook Golf Course (PB; Manalapan, NJ, USA; 40°32′ N, 74°31′ W), Howell Park Golf Course (HP; Farmingdale, NJ, USA; 40°18′ N, 74°19′ W), Preakness Hills Country Club (PH; Wayne, NJ, USA; 40°94′ N, 74°24′ W), and Rutgers Horticultural Farm No. 2 (HF; North Brunswick, NJ, USA; 40°47′ N, 74°42′ W). Turf stands at all sites consisted of mixtures of creeping bentgrass, *Agrostis stolonifera* L., and annual bluegrass, *Poa annua* L., mown at 8.3 to 12.7 mm height three to four times per week, with thatch thickness ranging from 3 to 6 mm and soil type ranging between loam and sandy loam. Experimental areas were managed by golf course staff and research farm staff, respectively, using the same procedures as the rest of the fairways, except that no insecticides were applied until after the experiment evaluation each year. To minimize potential differences in ABW densities across the sampling areas, in each experiment, the areas were divided into six equal-sized sub-plots, and for each extraction treatment, one sixth of the total number of cores was taken randomly from each sub-plot. The cores were placed in polyethylene bags by prospective extraction treatment and brought to the laboratory for processing. Before extraction, the bottom part of the cores was cut off, leaving the grass portion and about 2 cm of soil. ABW stages are rarely found below 1 cm soil depth [8].

Timing of sampling dates was determined using a combination of ABW larval sampling, indicator plant phenology, and growing degree day accumulation (base temperature of 10 °C, starting March 1; GDD_10_). Phenology of flowering dogwood, *Cornus florida* L., and hybrid Catawba rhododendron, *Rhododendron catawbiense* Michx., and GDD_10_ accumulation are proven indicators for timing of ABW larvicide applications in spring (e.g., [9,12]). GDD_10_ were recorded with a WatchDog Weather Tracker Model 300 (Spectrum Technologies, Aurora, IL, USA). Larval densities and phenology were determined weekly by taking 20 soil/sod cores (5.7 cm diameter × 4 cm depth) and extracting ABW stages (salt extraction as described below with cores torn into four pieces) from late blooming of *C. florida*, when larvae started feeding inside the grass stems, until past the blooming of *R. catawbiense* when most of the larvae were feeding externally. The larval instar average (Lavg) was calculated using the formula developed by Koppenhöfer et al. (2019, [12]), where n is the number of individuals recovered of a given instar; L1 through L5 are the five larval stages; Pu is the pupal stage; TA is the teneral adult; and N the total number of all stages recovered: Lavg = [nL1 × 1 + nL2 × 2 + nL3 × 3 + nL4 × 4 + nL5 × 5 + nPu × 6 + nTA × 7]/N.

### 2.2. Extraction Methods

For salt extraction, 200 g of table salt (approx. 98% NaCl) was placed into an 840 mL plastic cup (Comfy Package, Brooklyn, NY, USA). Then, an individual and undivided core was placed in the cup, and the cup was filled with lukewarm tap water to 500 mL, which dissolved most of the salt, creating a saturated saline solution. The water with the core material was vigorously stirred for 10 s immediately and again after 20 and 40 min. The stirring helped disintegrate the core material, thereby freeing ABW stages trapped in or under it and allowing them to float to the surface of the saline solution. The surface of the solution in the cups was checked for ABW stages just before the 20 and 40 min stirring and at 60 min after the first stirring. Any ABW stages found were transferred using a soft forceps into a well of a 24-well plate filled with 1 mL of 70% ethanol. After the last check for ABW stages in the cups, the saline solution was gradually transferred into a 150 × 25 mm Petri dish to check for any ABW stages remaining in it. These stages were then also transferred into the well with 70% ethanol for the corresponding cup. Once extractions were completed, the instar of the collected larvae was determined based on head capsule width [8] under a dissecting microscope, and all stages found were recorded.

For heat extraction with Berlese traps, 473 mL plastic cups (Dart Container Corp., Mason, MI, USA) were filled to about 2 cm height with soapy water (0.1% liquid dishwashing detergent). A funnel made from a 207 mL plastic coffee cup liner (SOLO Cup Company, Urbana, IL, USA) with the bottom cut away was fit into the plastic cup. Then, one individual core was placed grass side down into the funnel, and the Berlese traps were placed in an incubator kept at 32 °C. After 4 days, any ABW stages in the soapy water at the bottom of the Berlese traps were transferred into 70% ethanol, and ABW larval instars were determined, and all stages were recorded as was performed for the salt extractions. The remaining dried core was destructively examined for ABW stages that were separately collected in 70% ethanol, and stages were determined and recorded as above.

### 2.3. Experiments

Experiment 1 determined the effect of different degrees of processing cores before salt extraction on the extraction rate in comparison to heat extraction. Before salt extraction, cores were (1) left intact, (2) torn into four pieces of similar size, or (3) destructively searched for ABW stages. For simplicity, we will refer to these methods henceforth as (1) intact salt extraction, (2) four-piece salt extraction, and (3) destructive salt extraction. Larvae found during the pre-extraction processing were added to the well designated for the core. To determine any effects of average larval stage on the extraction efficiency, 30 cores per treatment, site, and sampling date were collected from PB and HP on 23 May 2011 and on 31 May 2011.

Experiment 2 determined the time required per core for each step of each extraction method used in the first experiment. For the salt extractions, the steps included the setup (preparing cups, adding salt, and adding water), the processing (no processing, tearing into four pieces, or destructively searching and collecting stages), and placing core material in cups, looking for larvae in cups, and looking for larvae in the large Petri dish. For the heat extraction, the steps included setting up (preparing Berlese traps and adding soapy water), placing the core in the trap, checking for larvae in the soapy water, and destructively searching the dried core. Two cores for each treatment were taken from each of the six subplots at PB (12 cores total per treatment). In the laboratory, the cores were extracted by two researchers (six cores per treatment each), and the time taken for each step was recorded. This was performed on 23 May 2011, and again on 31 May 2011.

Experiment 3 examined the effect of core size and the presence/absence of salt on the extraction rate. Treatments were (1) four-piece salt extraction as above, (2) salt extraction using smaller cores (3.5 cm × 4 cm depth) that were left intact, (3) four-piece salt extraction but without salt added to water, and (4) heat extraction. The smaller cores had been taken with a smaller diameter tubular turf plugger (Turf-Tec International, Tallahassee, FL, USA). To determine any effects of average larval stage on the extraction rate, 30 cores per treatment, site, and sampling date were collected from PB and HP on 20 May 2012 and on 31 May 2012.

Experiment 4 aimed at comparing the extraction rate for ABW populations with younger larval instar averages. Treatments were (1) four-piece salt extraction and (2) heat extraction. Thirty cores per treatment, site, and sampling date were taken from PH and HF on 20 May 2021, on 23 May 2021, and on 27 May 2021.

Experiment 5 tested whether the extraction rate and/or speed of heat extraction could be improved by splitting the cores into four pieces before extraction. Treatments were (1) four-piece salt extraction as above, (2) heat extraction as above, and (3) heat extraction as above except that the cores were torn into four pieces of similar size before being placed grass side down on the funnel. Thirty cores per treatment were taken from each of two fields at HF on 19 May 2025. To determine the speed of extraction for the heat extraction treatments, the soapy water in the bottom of the Berlese traps was checked 48, 72, and 96 h after placement of the cores, and any ABW stages found were transferred into a well of a 24-well plate filled with 1 mL of 70% ethanol.

### 2.4. Statistical Analysis

For Experiments 1 and 3–5, the relative extraction rate was calculated by dividing within each sampling date and sampling site the number of ABW stages (all larval stages, pupae, and teneral [not fully sclerotized] adults) extracted in each core by the average number found with four-piece salt extraction. This was performed to standardize the results and facilitate comparison among experiments, as the four-piece salt extraction was used in each experiment. Data were then normalized by square root or log (x + 1) transformation and subjected to ANOVA (software Statistix 10.0; Analytical Software, 2018), with sampling date, sampling site, and extraction method as factors, and interactions between factors were also examined. Means were separated using Tukey’s test. The larval instar average [12] was normalized as above and subjected to ANOVA with sampling date, sampling site, and extraction method as factors, and interactions between factors were also examined. Both analyses were conducted using the total number of ABW stages extracted in the heat extraction treatment and also using only the number recovered in the soapy water. In Experiment 2 (timing), data could not be normalized through transformations and were first analyzed by sampling date using the Kruskal–Wallis non-parametric test, followed by Dunn’s test for means separation. Since data from both sampling dates followed the same pattern, the data from both sampling dates were combined for analysis. Differences among means were considered significant at *p* < 0.05. Means ± SEM are presented.

## 3. Results

**Figure 1:**
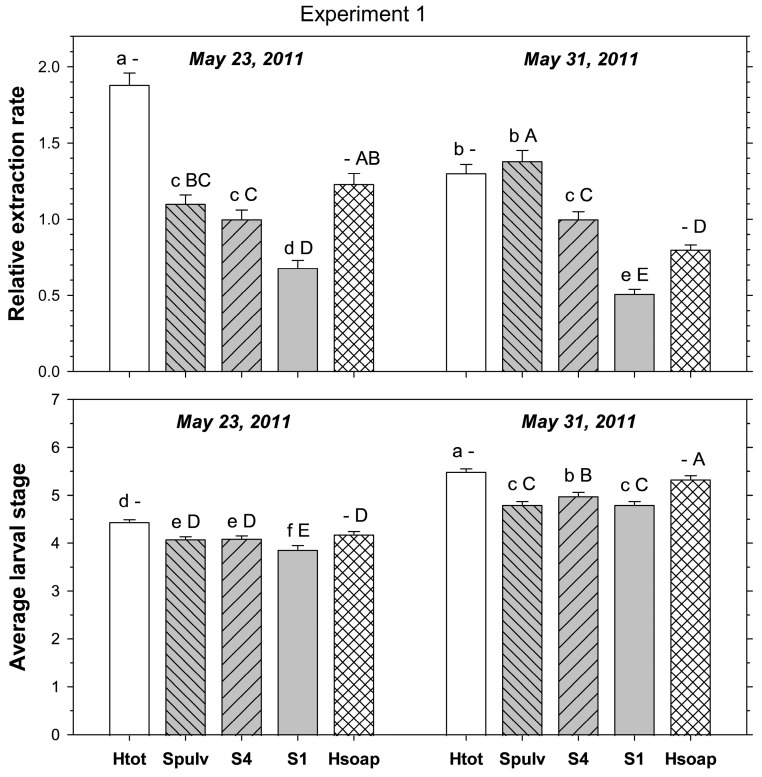
Extraction from turf cores of mature *Listronotus maculicollis* larval populations using destructive (Spulv), four-piece (S4), and intact core (S1) salt extraction compared to heat extraction, including (Htot) or excluding (Hsoap) larvae found in the desiccated cores left after heat extraction (see text for details). Top plate shows extraction rate (relative to four-piece salt extraction = S4), bottom plate shows the average larval stage extracted. Lower case letters above bars indicate significant differences among extraction methods excluding Hsoap; upper case letters indicate significant differences among methods excluding Htot (*p* < 0.05).

In the first experiment, relative extraction rate was significantly affected by extraction method (F_3, 479_ = 103.34; *p* < 0.001) and sampling date (F_1, 479_ = 6.02; *p* < 0.05); method and date interacted significantly (F_3, 479_ = 13.13; *p* < 0.001). In samples taken on 23 May 2011, heat extracted more ABW stages than destructive and four-piece salt extractions, which both extracted more stages than intact salt extraction (Figure 1). In samples taken on 31 May 2011, heat and destructive salt extraction extracted more stages than four-piece salt extraction, with intact salt extraction being the least effective method.

When salt extraction methods were compared to the heat extraction excluding stages found in the desiccated cores, relative extraction rate was significantly affected by extraction method (F_3, 479_ = 49.51; *p* < 0.001) and sampling date (F_1, 479_ = 5.17; *p* < 0.05); method and date interacted significantly (F_3, 479_ = 11.94; *p* < 0.001). In samples taken on 23 May 2011, heat extracted more stages than four-piece and intact salt extractions, and destructive salt extraction extracted more stages than intact salt extraction (Figure 1). In samples taken on 31 May 2011, heat extracted fewer stages than destructive and four-piece salt extractions, but more stages than intact salt extraction, and destructive salt extraction was the most efficient method.

The average larval stage of the extracted ABW was significantly affected by the extraction method (F_3, 479_ = 43.31; *p* < 0.001) and sampling date (F_1, 479_ = 456.84; *p* < 0.001); method and date interacted significantly (F_3, 479_ = 2.78; *p* < 0.05). For each sampling method, the average stage was more advanced in the later than the earlier sample (Figure 1). In samples taken on 23 May 2011, the average stage was more advanced with heat extraction than with destructive and four-piece salt extractions, which were more advanced than intact salt extraction. In samples taken on 31 May 2011, the average stage was more advanced in the heat extraction than in the four-piece salt extraction, which had more advanced stages than destructive and intact core salt extractions.

When salt extraction methods were compared to heat extraction excluding stages found in the desiccated cores, the average stage was significantly affected by extraction method (F_3, 479_ = 17.19; *p* < 0.001) and sampling date (F_1, 479_ = 445.49; *p* < 0.001); method and date interacted significantly (F_3, 479_ = 3.85; *p* < 0.01). In samples taken on 23 May 2011, the average stage in the heat extraction did not differ significantly from that found with destructive and four-piece extractions; all three had more advanced stage averages than intact salt extraction (Figure 1). In samples taken on 31 May 2011, heat extraction had a higher larval average than four-piece salt extraction, which had a higher average than destructive and intact salt extractions.

**Figure 2:**
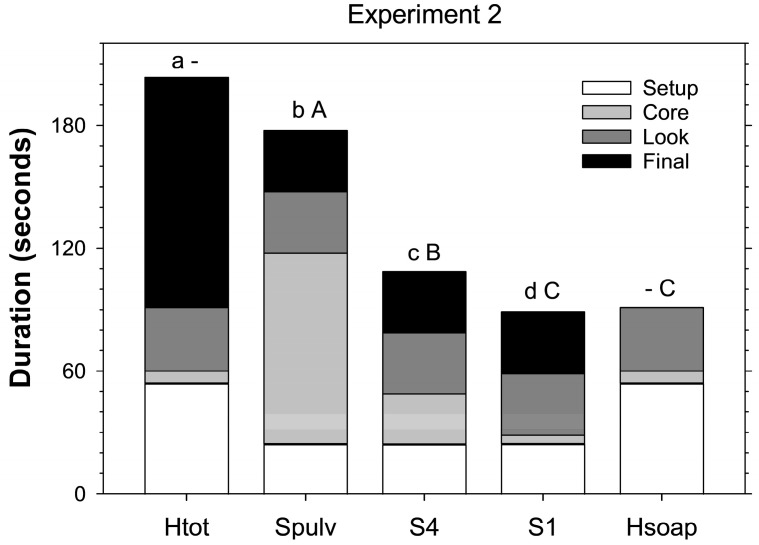
Time required per core for each step of the four extraction methods for *Listronotus maculicollis* larvae. For the salt extractions (S-), the steps included setup (setup: preparing cups, adding salt, adding water), the processing of the core (core: no processing (S1), tearing into four pieces (S4), or destructively searching (Spulv) and collecting stages) and placing of core material in cups, looking for larvae in cups (Look), and looking for larvae in the large Petri dish (final) (see text for details). For the heat extraction, the steps included setup (setup: preparing Berlese traps and adding soapy water), placing the core on the trap (core), checking for larvae in the soapy water (look), and destructively searching the dried core (final). Lower case letters above bars indicate significant differences among extraction methods excluding Hsoap; upper case letters indicate significant differences among methods excluding Htot (*p* < 0.05).

In the second experiment, total time required to extract a sample core differed significantly between extraction methods (F_3, 95_ = 323.89; *p* < 0.001) and was the longest for heat extraction (203.3 ± 2.6 s including searching through the desiccated core), followed closely by destructive salt extraction (177.5 ± 2.3), then four-piece salt extraction (108.5 ± 1.3), and was the least for intact salt extraction (88.8 ± 1.4) (Figure 2). Heat extraction without going through the desiccated core took less than half as many seconds (91.0 ± 1.0) as when including the core and took significantly less time than four-piece and destructive salt extraction (F_3, 95_ = 159.09; *p* < 0.001) (Figure 2).

**Figure 3:**
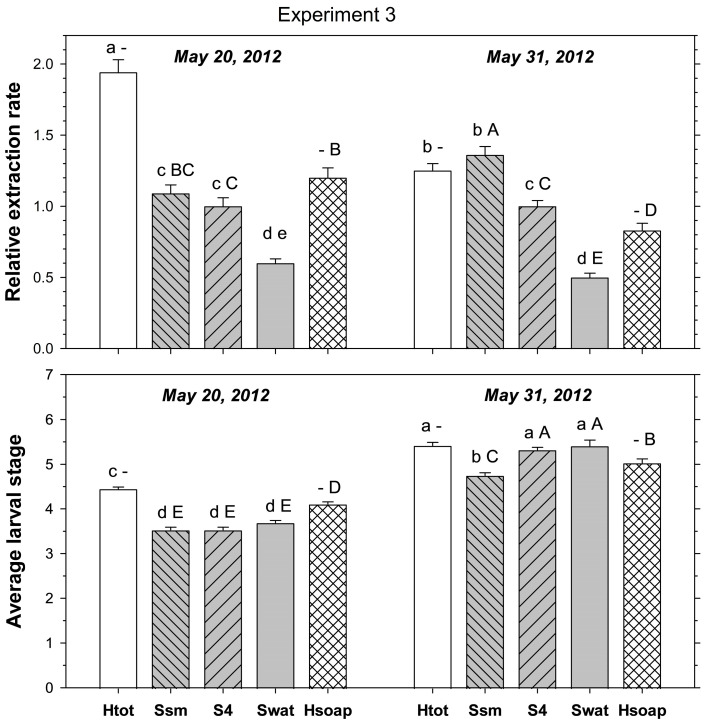
Extraction from turf cores of mature *Listronotus maculicollis* larval populations using small core (Ssm) and four-piece (S4) salt extraction compared to saltless (Swat) four-piece extraction and heat extraction, including (Htot) or excluding (Hsoap) larvae found in the desiccated cores left after heat extraction (see text for details). Top plate shows extraction rate (relative to four-piece salt extraction = S4), bottom plate shows average larval stage extracted. Lower case letters above bars indicate significant differences among extraction methods, excluding Hsoap; upper case letters indicate significant differences among methods excluding Htot (*p* < 0.05).

In the third experiment, relative extraction rate was significantly affected by extraction method (F_3, 479_ = 151.47; *p* < 0.001) and sampling date (F_1, 479_ = 8.25; *p* < 0.01); method and date interacted significantly (F_3, 479_ = 22.76; *p* < 0.001). In samples taken on 20 May 2012, heat extracted more ABW stages than small core and four-piece salt extractions, which both extracted more stages than saltless four-piece extraction (Figure 3). In samples taken on 31 May 2012, heat and small core salt extraction extracted more stages than four-piece salt extraction, with saltless four-piece extraction being the least effective method.

When salt extraction methods were compared to the heat extraction excluding stages found in the desiccated cores, relative extraction rate was significantly affected by extraction method (F_3, 479_ = 72.37; *p* < 0.001) but not by sampling date (F_1, 479_ = 2.60; *p* = 0.107); method and date interacted significantly (F_3, 479_ = 13.38; *p* < 0.001). In samples taken on 20 May 2012, heat extracted more stages than four-piece salt extraction and saltless four-piece extractions, but not more than small core salt extraction (Figure 1). In samples taken on 31 May 2012, heat extracted fewer stages than small core and four-piece salt extraction, but more than saltless four-piece extraction.

The average larval stage of the extracted ABW was significantly affected by the extraction method (F_3, 479_ = 151.47; *p* < 0.001) and sampling date (F_1, 479_ = 4801.63; *p* < 0.001); method and date interacted significantly (F_3, 479_ = 15.38; *p* < 0.001). For each sampling method, the average stage was more advanced in the later than the earlier sample (Figure 1). In samples taken on 20 May 2012, the average stage was more advanced in heat extraction than in all other methods; the other methods did not differ amongst themselves. In samples taken on 31 May 2012, the average stage was more advanced in heat extraction, four-piece salt extraction, and saltless four-piece extraction than in small core salt extraction.

When salt extraction methods were compared to heat extraction excluding stages found in the desiccated cores, the average stage was significantly affected by extraction method (F_3, 479_ = 123.21; *p* < 0.001) and sampling date (F_1, 479_ = 77.58; *p* < 0.001); method and date interacted significantly (F_3, 479_ = 24.16; *p* < 0.010). In samples taken on 20 May 2012, the average stage in heat extraction was more advanced than in all other methods; the other methods did not differ amongst themselves (Figure 1). In samples taken on 31 May 2012, heat extraction had a less advanced larval average than four-piece salt extraction and saltless four-piece extraction, but was more advanced than with small core salt extraction.

**Figure 4:**
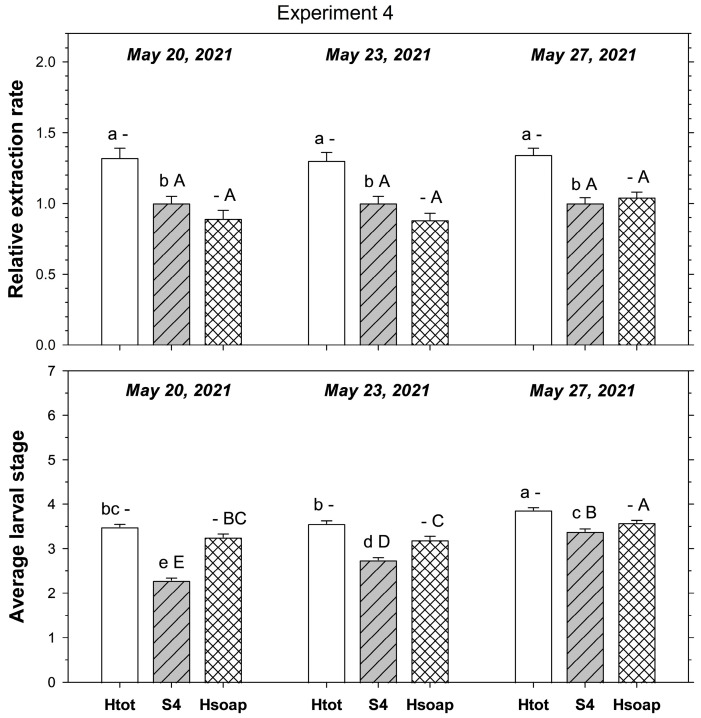
Extraction from turf cores of young *Listronotus maculicollis* larval populations comparing four-piece (S4) salt extraction to heat extraction, including (Htot) or excluding (Hsoap) larvae found in the desiccated cores left after heat extraction (see text for details). Top plate shows extraction rate (relative to four-piece salt extraction = S4), bottom plate shows average larval stage extracted. Lower case letters above bars indicate significant differences among extraction methods excluding Hsoap; upper case letters indicate significant differences among methods excluding Htot (*p* < 0.05).

In the fourth experiment, relative extraction rate was significantly affected by extraction method (F_1,359_ = 56.24; *p* < 0.001) but not by sampling date (F_2, 359_ = 0.24; *p* = 0.788); method and date did not interact (F_2, 359_ = 0.08; *p* = 0.919). On all sampling dates, heat extracted more ABW stages than four-piece salt extraction (Figure 4). When four-piece salt extraction was compared to the heat extraction excluding stages found in the desiccated cores, relative extraction rate was not significantly affected by extraction method (F_1,3579_ = 3.68; *p* = 0.056) and sampling date (F_2, 359_ = 2.30; *p* = 0.102); method and date did not interact (F_2, 359_ = 1.83; *p* = 0.162).

The average larval stage of the extracted ABW was significantly affected by extraction method (F_1, 359_ = 357.99; *p* < 0.001) and sampling date (F_2, 359_ = 94.79; *p* < 0.001); method and date interacted significantly (F_2,359_ = 22.04; *p* < 0.001). The average stage was more advanced in heat extraction than four-piece salt extraction on each sampling date, but the difference decreased from the first to the second to the third sampling date.

When salt extraction methods were compared to the heat extraction excluding stages found in the desiccated cores, the average stage was significantly affected by extraction method (F_1, 359_ = 123.21; *p* < 0.001) and sampling date (F_2, 359_ = 77.58; *p* < 0.001); method and date interacted significantly (F_2, 359_ = 24.16; *p* < 0.01). As there was no large difference between total heat extraction and heat extraction without the desiccated core, the average stage was also higher in the heat extraction than in the four-piece salt extraction on each sampling date, with the difference decreasing from the first to the second to the third sampling date.

**Figure 5:**
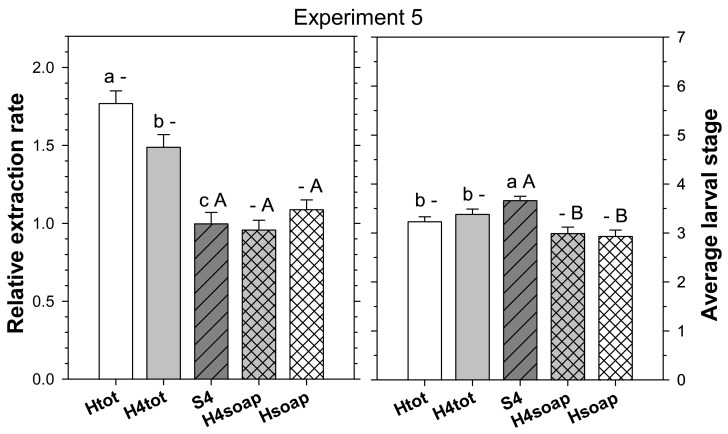
Extraction from turf cores of young *Listronotus maculicollis* larval populations comparing four-piece (S4) salt extraction to heat extraction with cores kept intact (H) or split into four pieces (H4). Heat extraction data include (Htot/H4tot) or exclude (Hsoap/H4soap) larvae found in the desiccated cores left after heat extraction (see text for details). Top plate shows extraction rate (relative to four-piece salt extraction = S4); bottom plate shows average larval stage extracted. Lower case letters above bars indicate significant differences among extraction methods excluding Hsoap and H4 soap; upper case letters indicate significant differences among methods excluding Htot and H4tot (*p* < 0.05).

In the fifth experiment, relative extraction rate was significantly affected by extraction method (F_2,179_ = 30.06; *p* < 0.001) but not by site (F_1, 179_ = 0.00; *p* = 0.947); method and site did not interact (F_2, 179_ = 2.10; *p* = 0.126). Heat extraction using intact cores extracted more ABW stages than heat extraction with cores split into four pieces, which extracted more ABW stages than four-piece salt extraction (Figure 5). When four-piece salt extraction was compared to the heat extraction excluding stages found in the desiccated cores, relative extraction efficiency was not significantly affected by extraction method (F_2,179_ = 1.56; *p* = 0.214) and site (F_1, 179_ = 0.85; *p* = 0.357) (Figure 5).

The average larval stage of the extracted ABW, whether including or excluding the stages found in the desiccated core, was significantly affected by extraction method (F_2, 179_ ≥ 5.51; *p* < 0.01) and site (F_1, 179_ ≥ 6.10; *p* < 0.05), and method and site did not interact (F_2, 179_ ≤ 1.19; *p* ≥ 0.308). The average stage was more advanced in four-piece salt extraction than in both heat extraction methods (Figure 5).

The cumulative number of ABW stages found in the soap solution of the heat extractions was not significantly affected by method after 2, 3, and 4 days (F_1, 119_ ≤ 3.12; *p* ≥ 0.080). The cumulative extraction rate relative to the four-piece salt extraction after 2, 3, and 4 days was 0.70 ± 0.04, 0.88 ± 0.05, and 1.09 ± 0.06, respectively, for heat extraction using intact cores and 0.65 ± 0.03, 0.88 ± 0.05, and 0.96 ± 0.06, respectively, for heat extraction with cores split into four pieces. The cumulative average larval stage was more advanced in the heat extraction method with cores split into four pieces after 2 days (F_1, 119_ = 9.20; *p* = 0.003), but there was no significant difference after 3 and 4 days (F_1, 119_ ≤ 0.13; *p* ≥ 0.717). The cumulative average larval stage after 2, 3, and 4 days was 3.17 ± 0.12, 3.05 ± 0.11, and 2.94 ± 0.12, respectively, for heat extraction using intact cores and 3.26 ± 0.12, 3.11 ± 0.13, and 3.00 ± 0.12, respectively, for the heat extraction with cores split into four pieces.

## 4. Discussion

Our research showed significant differences in the extraction rate for ABW larvae and time requirements among variants of the salt extraction method and between the salt extraction and heat extraction methods. Moreover, the extraction rate and relative rate among methods were also affected by the average larval stage distribution at the time of extraction initiation.

The core submersion method clearly required a saturated saline solution, as water alone extracted around 45% fewer larvae than saturated saline. The extraction rate of the saltless four-piece method (relative to the rate of the four-piece salt extraction in the same experiments on the same sampling date) was not significantly affected by the average larval stage present and was 60% and 50% when the average larval stage was L3.69 and L5.41, respectively.

Among the salt extraction variants using the standard size cores (5.7 cm diameter), the four-piece method seemed to be the best compromise between extraction rate and time requirement. In comparison to it, the intact-core method extracted 40% fewer larvae while only taking 18% less time, whereas the destructive searching method extracted 24% more larvae but required 64% more time. Even better than the four-piece method was the method using the smaller cores (3.5 cm diam), which took 18% less time and extracted 23% more larvae. However, this method requires more time in the field as more cores need to be taken to compensate for the smaller sample size and, accordingly, more holes need to be filled afterwards. But the larger number of cores allows for a better spread of samples throughout a sampling area, and taking smaller cores is less obtrusive to the turf. Larval stage averages generally did not differ significantly between salt extraction variants, except that stages were younger with the small cores on the late sampling dates in Experiment 3. However, there seems to be no logical reason for a difference in larval stage averages between the small-core method and the four-piece method.

Heat extraction, including destructively searching the desiccated core, had by far the highest extraction rate but also took the greatest amount of time. Compared to the four-piece salt extraction, it extracted 60% more larvae but required 87% more time. Without destructively searching the desiccated core, heat extracted as many larvae as four-piece salt extraction and required 16% less time. Heat extraction, therefore, would seem like the method of choice, whether the higher extraction is required (including destructively searching the desiccated core) or the higher efficiency (without desiccated core).

Heat extraction, however, has two drawbacks, particularly for practitioners like consultants or golf course superintendents. First, it takes at least three additional days to obtain the results, which is a problem when the findings call for an insecticide application as soon as possible. In addition, heat extraction requires a space such as an incubator that can be kept warm enough and can hold a good number of extraction cups. This might be limiting for a superintendent, but feasible for consultants or laboratories conducting a limited amount of monitoring. The evaluation of large experiments, let alone several such experiments overlapping in time, or extensive monitoring of multiple sites, would require large incubators, often several, or small rooms, and this could be limiting even for a research laboratory. Another minor drawback of heat extraction, especially when including the stages found in the desiccated cores, is that the stages advance significantly and likely faster than they would under field conditions.

Our last experiment sought to address the above limitation of heat extraction. However, splitting the cores into four pieces did not increase extraction efficiency nor speed thereof, likely because with the small funnels used in our study, the core pieces ended up partially stacked on top of each other. This may have forced larvae exiting the higher pieces to travel across several pieces. Hence, heat extraction should be conducted using intact cores left on the funnels for at least 3 days.

Overall, our study shows the advantages and drawbacks of the various extraction methods tested. The method of choice for ABW larval extractions will depend on whether the sampler prefers high extraction rates or less labor and quicker results. If enough space is available or only a limited number of cores need to be extracted and waiting for a few days to obtain results is not a problem, then heat extraction with intact pieces for three or, better, four days would be the method of choice. It provides the highest extraction rate when also inspecting the desiccated cores, or the least amount of labor, with a still good extraction rate when not inspecting the desiccated cores. If, however, there is not enough space and results need to be obtained quickly, then four-piece salt extraction is the best method.

## Data Availability

The data presented in this study are available upon request from the corresponding author.
